# Sensing and 3D Mapping of Soil Compaction

**DOI:** 10.3390/s8053447

**Published:** 2008-05-26

**Authors:** Yücel Tekin, Basri Kul, Rasim Okursoy

**Affiliations:** 1 Uludağ University, Vocational School of Technical Sciences, 16059 Görükle Campus, Bursa – Turkey; E-mail: basrikul@uludag.edu.tr; 2 Uludağ University, Faculty of Agriculture, Department of Agricultural Machinery, 16059 Görükle Campus, Bursa – Turkey; E-mail: okursoy@uludag.edu.tr

**Keywords:** Soil compaction, Soil mapping, Penetration resistance, GPS

## Abstract

Soil compaction is an important physical limiting factor for the root growth and plant emergence and is one of the major causes for reduced crop yield worldwide. The objective of this study was to generate 2D/3D soil compaction maps for different depth layers of the soil. To do so, a soil penetrometer was designed, which was mounted on the three-point hitch of an agricultural tractor, consisting of a mechanical system, data acquisition system (DAS), and 2D/3D imaging and analysis software. The system was successfully tested in field conditions, measuring soil penetration resistances as a function of depth from 0 to 40 cm at 1 cm intervals. The software allows user to either tabulate the measured quantities or generate maps as soon as data collection has been terminated. The system may also incorporate GPS data to create geo-referenced soil maps. The software enables the user to graph penetration resistances at a specified coordinate. Alternately, soil compaction maps could be generated using data collected from multiple coordinates. The data could be automatically stratified to determine soil compaction distribution at different layers of 5, 10,.…, 40 cm depths. It was concluded that the system tested in this study could be used to assess the soil compaction at topsoil and the randomly distributed hardpan formations just below the common tillage depths, enabling visualization of spatial variability through the imaging software.

## 1. Introduction

Soil examination techniques in the field have been widely used for many centuries. They are used for evaluating the quality of land, for studies of soil genesis, soil compaction, erosion control, and for tillage management [1]. Soil compaction is an important physical limiting factor for the root growth and plant emergence, decreasing crop production worldwide. It is often caused by heavy axle loads of agricultural machines such as tractors and self-propelled harvesters as well as other equipment used in agro-technical operations during planting and vegetation. Soil compaction may significantly debilitate the production capacity of soil by reducing porosity, creating obstacles to air, water, nutrient movements and root penetration [2, 3]. In addition, soil compaction reduces rate of leaf appearance and ground cover expansion, shortened canopy cover duration and restricted light interception, which combined to reduce tuber yield [4]. Reductions in grain yield attributable to soil compaction for several climate and crops in a wide range of soils from sands to heavy clays [5-9]. Moreover, the subsoil becomes a compacted soil layer which prevents water from infiltrating into deeper layers, resulting in reduced porosity at topsoil and decreased yields [10]. Therefore, researchers are interested in focusing on subsoil compaction and the methods of tillage to control the compacted layer [11, 12]. Although some researchers show no statistical conclusion could be drawn on the effect of subsoiling on crop yields [13], the soil compaction should be mapping to reduce draft force. Site-specific subsoiling resulted in 59% and 35% reduced draft force in the shallow depth hardpan plots (25 cm) and medium depth hardpan plots (35 cm), respectively, compared to uniform deep subsoiling conducted at 45 cm depth reported by Raper et. al., [14].

Soil compaction is commonly expressed as penetration resistance (PR) measured by a soil cone penetrometer. A simple penetrometer is a penetration rod having a conical tip with a force sensor, which may be a strain-gauge or piezoelectric load cell [15]. PR is defined as the penetration force divided by a standard cone base area during the penetration of the soil with a standard soil cone penetrometer at a constant penetration rate [16]. The standard penetrometer cone has a 30° cone tip angle and a 2.54 mm base diameter. The penetration rate is also standardized as 30 mm s^-1^. Although there is more than one method to assess soil compaction, the most convenient method for most researchers in field conditions for monitoring and assessing soil compaction has long been using handheld penetrometers. Thus, the accuracy of PR measurements is highly related to the ability to maintain a constant probe speed.

Penetration resistance measurements are usually related to soil moisture content and bulk density. Therefore, researchers developed models to relate soil compaction to dry bulk density and soil moisture content [17-19]. Geostatistics in a study revealed that the most variable soil property was PR whereas the least varying parameter was the soil bulk density [20]. Geostatistical techniques can also be used to interpolate PR data to produce three-dimensional maps. Some studies show that PR randomly varies across the fields [21]. In practice, however, collecting enough point data with high accuracy across a whole field, so that geo-referenced soil compaction maps can be produced, requires long time and excessive efforts. Recently, studies focus more on the use of hydraulically driven penetrometers with electronic kits for mapping soil compaction [3]. The soil compaction maps allow researchers and farmers to pin out where exactly the soil compaction occurs [3]. Thus the farmer can observe how soil compaction varies at different locations and depths across the field. This information is critical in decision making process for site-specific applications such as variable deep tillage to benefit from increased timeliness and reduced management costs.

The objective of this study is to describe the hardware and software that have been developed, and then testing the system to justify the system's applicability. This system instantaneously generates 2D and 3D soil compaction maps through a data collection and mapping software developed specifically for this study.

## 2. Materials and Methods

### 2.1. System Design

**Figure 1.**
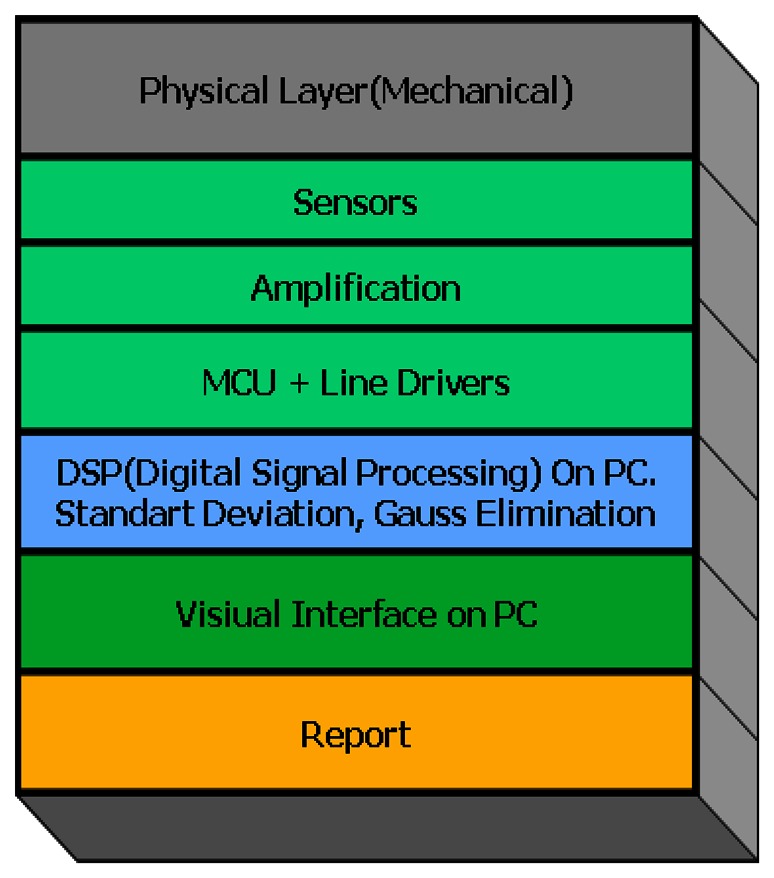
Structure of penetration resistance measurement and mapping system.

The three dimensional soil compaction mapping procedure consists of 3 layers: Physical layer consisting of mechanical parts and sensors, data acquisition system (DAS) layer controlled by a micro processor, and 2D/3D imaging and analysis software layer. Physical layer includes a standard penetrometer driven by hydraulic system of tractor, sensors and mechanical controls. DAS layer includes MCU controlled data collection and security controls. The 2D/3D imaging and analysis layer includes required imaging, analyzing and reporting. Embedded analyzing is ensured instead of analyzing the results of point measurements as seen in similar studies and real time assessment in designing system. The general structure of the system that was designed specifically for measuring soil compaction and real-time mapping is showed in Figure 1.

#### 2.1.1. Physical layer

**Figure 2.**
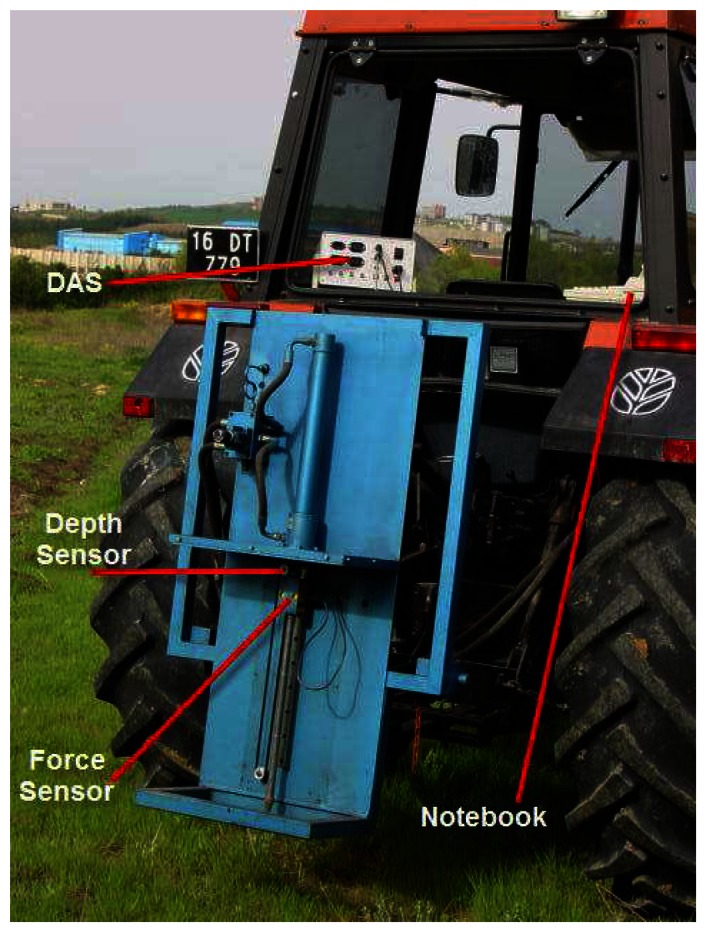
Hydraulically driven penetrometer mounted on the hitch of the tractor

The PR measurements with a tractor hydraulics driven penetrometer were used for soil compaction mapping [22]. The electronic penetrometer mounted on the tractor consists of a load-cell, a depth sensor, DAS and a notebook (Figure 2). The hydraulic cylinder is powered by timing gears and couplings, which are located in a hydraulic pump. The oil flow of the pump depends on the tractor engine speed and at 1400 rpm oil flow through the penetrometer hydraulic cylinder provides the penetrometer tip a constant and standard penetration rate. Because the standard penetration rate of the penetrometer is 30 mm per second and the maximum penetration depth is 40cm, taking data from a location takes 13.3 seconds, which is a very short time. For a standard penetration rate, oil flow for the cylinder is 2.26 L min^-1^ and the oil pressure for the cylinder is 30.8 bars. The oil flow is controlled by a two-way check valve which has a maximum oil pressure of 210 bars and maximum flow rate of 1.54 L min^-1^ [23].

The soil depth sensor was made using multiturn resistance (trimpot), which is a roller pulley with a belt drive. When the hydraulic piston pushes the penetrometer rod, the cone starts to penetrate the soil at a constant speed. When the penetration starts, an elastic belt turns the small pulley that generates an electronic signal. The force sensor is an S-type load cell placed between the piston and the penetrometer rod. The load cell of the force transducer is a Wheatstone bridge circuit that needs 5V to excite and to reach an electronic balance. The full scale measurement interval of the circuit is 20mV, which corresponds to a 500 kg load with a measurement sensitivity of 0.007 kg. The calibration of the load cell was performed by exerting loads in laboratory condition. Calibration result of the force sensor shows that there exists an exact linearity (R^2^ = 0.999973) between the output of the force sensor and the exerted force ranging from 0 to 500 kg. When a penetration force is exerted on the cone, the electronic balance of the bridge circuit changes and the circuit produces output signals that are amplified and converted into digital signal for calibration [24].

Previous researches showed that hydraulically driven penetrometer could be used for acquiring accurate soil PR values. The difference of this study is to develop more quick data acquisition with USB port and to generate 2D and 3D soil compaction maps through these data.

#### 2.1.2. Data acquisition system (DAS)

**Figure 3.**
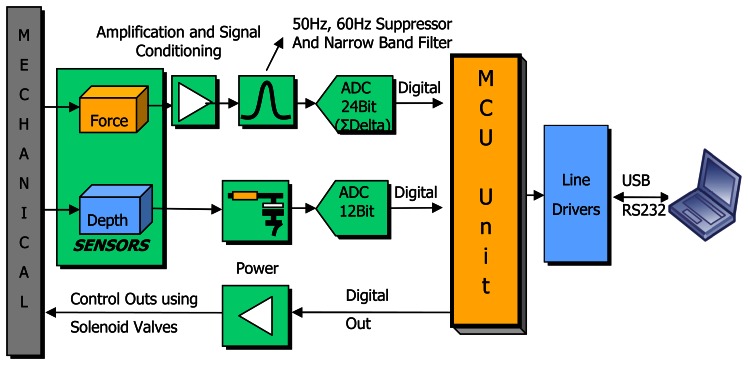
Block diagram of DAS.

The DAS was designed to process the signals acquired from the load-cell and depth sensor and to control the mechanical system (Figure 3). Signals taken from the load cell are converted into digital signal through an amplifier, a 50-60 Hz notch filter and a 24 bit sigma-delta ADC. The signals received from depth sensor are filtered from the noises from the tractor and white noise with use of a low-pass filter (LPF). Digital inputs are used for controlling mechanical system able to intervene in emergency situations. Total measurement capacity is designed for 10^6^ sampling data.

Data collection unit pulls back penetrometer rod automatically in overload cases and consequently prevents load-cell from damaging and also prevents from inaccurate measurements. The measurement depth can be set by the operator. DAS triggers all procedures with the commands from PC software. These are triggering and stopping the measurement, maximum depth, maximum force, calibration and time interval commands.

#### 2.1.3. PR 2D/3D –mapping and analysis software

**Figure 4.**
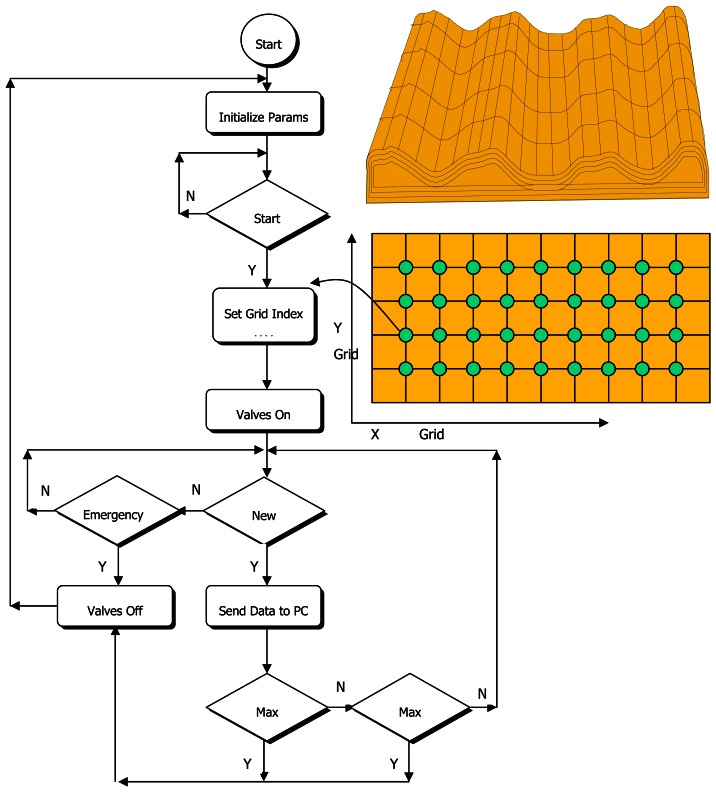
Flow chart of software of DAS.

Abbreviated flow chart of the Soil Compaction Measurement Software (SCMS) is shown in Figure 4. The software runs in MS windows XP medium and carries out the following functions:

- Definition of the field surface, determination of reference points with GPS
- Measurement of related attributes
- Digital filtering with DSP (Digital Signal Processing) procedures
- Creating visual data for one cm depth interval with the help of 2D/3D Digital Image Processing (DIP) methods
- Reporting (export for Excel and MATLAB)

The area within the specified coordinates may be informed to the software by gridding in desired intervals according to the nature of the soil. Left bottom is assigned to be the reference so that the x-y coordinate system could be used easily. The following control functions were used in the SCMS:

- Approval of the operator for the measurement in each cell: This procedure is important especially for proper alignment of the probe and the cone with the soil surface, allowing instant visual checking for hard objects before the cone starts penetrating the soil.
- Interrupting the recording process when the cone encounters hard matters such as stones that could cause erroneous measurements.
- Averaging multiple measurements for each datum to reduce standard deviation in measured quantities and for eliminating outliers.
- Scaling force sensor data taken from different depths of the field according to the same color scale.

The SCMS consists of Free Run, Measurement, Compute, Setup, and GPS menus. Free Run is used when measurement is conducted at a point without recording. Any point may be monitored to make sure that the probe is at a pre-determined or desired location. Data that were obtained from this menu, however, are not used for mapping. Measurement menu includes start, stop, load, save, and their configurations. Also, it ensures the input of x-y coordinates of grid points if GPS is not used.

Data are analyzed to perform 2D/3D mapping in Compute menu. In this part, data from the force sensor may be filtered, if desired, by selecting one of the filtering arrangements, namely 50 Hz infinite impulse response (IIR) filter, 50 Hz LPF and averaging filter. PR values are calculated depending on the measured force and cone area, and eventually expressed in kPa (eq. 1).

(1) $$PR=\frac{\text{Force}\left(N\right)}{\text{Cone}\_\text{Area}\left(m^{2}\right)}$$

Interpretation of the variation of soil compaction throughout the field is easier via graphical representation and by creating a color scale based on the range of measured values. 2D and 3D graphs are generated after field measurements have been completed. Scaling is done in such a way that blue shows the lowest compaction range and violet shows the highest compaction in the image. Numerical values corresponding to each color are also given in 2D and 3D graphics. These data can also be tabulated on the screen without generating the compaction maps. 2D functions used in the SCMS are as follows:

- Displaying measurements at cm intervals: PR values for each cm depth are displayed on 2 dimensional graphics according to the color scale. Depth up to 40 cm can be examined by this method.
- Calculating overall mean at a location: PR value of a cell is determined by calculating the mean of all measurements at each location on each grid (eq. 2).
  (2) $$\bar{PR}=\frac{1}{N}\sum_{i=1}^{N}PR_{i}$$
- Derivation for determining variations: This function analyzes PR variations in a grid with respect to the neighboring grids. Thus, variation slope of compaction can be displayed on a graph. To display variations of PR, directional derivative with the Laplacian filters are used such as *A*[*PR_x,y_*] diagonal (eq. 3), *B*[*PR_x,y_*] horizontal, *C*[*PR_x,y_*] (eq. 4), vertical (eq. 5). Magnitude of PR values shown as X in eq. 6 are applied and calculated by vertical, horizontal and diagonal Laplacian filters [25].

(3) $$A\left[PR_{x,y}\right]=\frac{1}{k}\left[\begin{array}{ccc} 2 & 1 & 0\\ 1 & 0 & -1\\ 0 & -1 & -2 \end{array}\right]*\left[PR_{x,y}\right]$$

(4) $$B\left[PR_{x,y}\right]=\frac{1}{k}\left[\begin{array}{ccc} 1 & 0 & -1\\ 2 & 0 & -2\\ 1 & 0 & -1 \end{array}\right]*\left[PR_{x,y}\right]$$

(5) $$C\left[PR_{x,y}\right]=\frac{1}{k}\left[\begin{array}{ccc} 1 & 2 & 1\\ 0 & 0 & 0\\ -1 & -2 & -1 \end{array}\right]*\left[PR_{x,y}\right]$$

(6) $$X=\sqrt{A^{2}+B^{2}+C^{2}}$$

3D mapping is similar to 2D, incorporating the latitude in the graphs thereby displaying measurements at cm intervals in 3D.

Outliers caused by stone and hard objects from penetrometer should be eliminated to reduce measurement errors. Potential outliers were eliminated in point measurements through the examination of standard deviations. The standard deviation and the mean were calculated using eqs. 7-8 and the data outside ±2σ range were ignored (eq. 9).

(7) $$\sigma =\sqrt{\frac{1}{N}\left(\sum_{i=1}^{N}x_{i}^{2}-N_{x}^{-2}\right)}$$

(8) $$\bar{x}=\frac{1}{N}\sum_{i=1}^{N}x_{i}$$

(9) $$x_{i}>\bar{x}+2\sigma ,\;x_{i}<\bar{x}-2\sigma$$

### 2.2. Experimental site

In November 2007 the PR tests were carried out in a clay soil (clay 58%, sand %24, silt 18) in heavy conditions at the Uludag University Research Farm, in Bursa-Turkey (longitude 28:98 E; latitude 40:22 N, altitude 122.5 m). The measurements were done following two year rotation of rapeseed. The average soil moisture content (dry base) was determined to be 24.79% with three replicates.

### 2.3. Data collection

The research field was selected as a flat and rectangular shape (27*39 m). The field was divided into plots with 3*2 m and marked for penetration measurements. The coordinates of plots are indicated by lap and index numbers on the SCMS. The DAS sampled 40 data for each centimeter. A total 180 point of PR measurements was obtained for soil compaction mapping. Then, SCM compiled the measured data for 2D/3D soil compaction mapping at 5 cm intervals from 0-40 cm.

## 3. Results and Discussion

**Figure 5.**
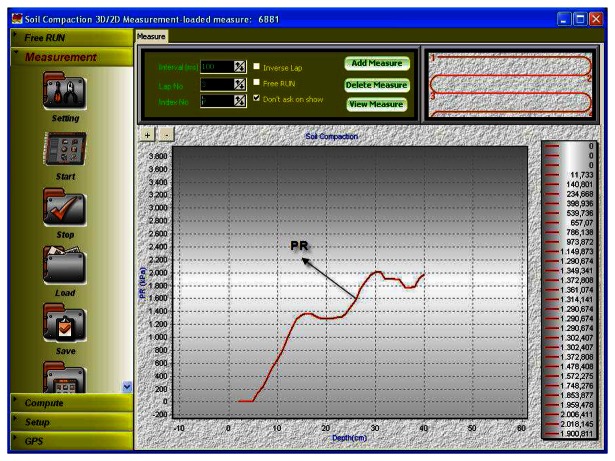
Measurement menu of the SCMS and plot of measured PR values for all layers.

**Figure 6.**
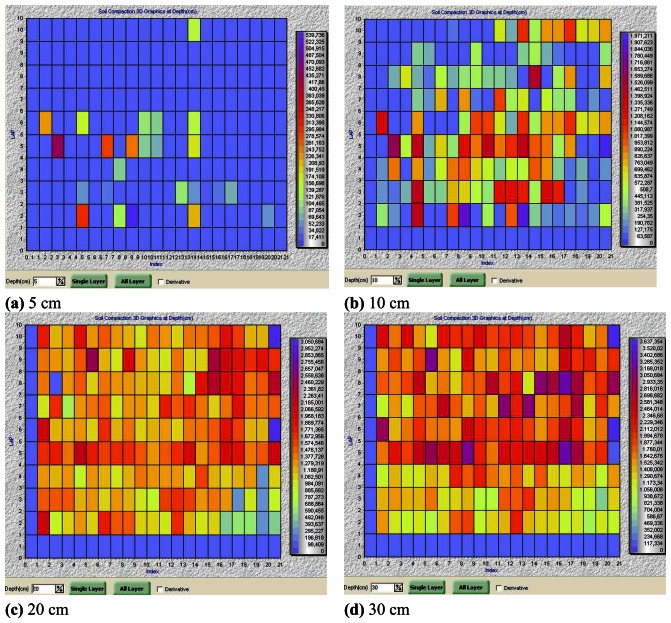
2D soil compaction mapping for various soil layers.

2D representations of measured penetration resistance values can be seen in Figures. 5 and 6. Figure 5 depicts measurement depth in x-axis and penetration resistance in y-axis. The PR values keep increasing at a constant rate up to 15 cm whereas the measured values do not vary significantly between 15 and 23 cm, followed by a second rise up to 30 centimeters. Similar trends could be observed in all cultivated soils in that hardpan occurs below the tillage depth which is usually at about 30-35 cm. The small fluctuations without a rise in measurements from 15 to 25 cm may be explained by the fact that soil was tilled using a primary tillage equipment that loosened the topsoil. The increasing PR values from 0 to 15 cm were probably due to the fact that the field traffic followed by tillage operation caused compaction at the topsoil albeit loosened during tillage operations. The soil bulk density also increases with increasing depth, resulting in reduced porosity and hence increased compaction at these layers.

An average PR of 2 MPa is usually accepted to be the threshold penetration resistance for most crops, which impedes root growth and causes reduced yield. It can be observed in Figure 5 that the soil compaction was close to the limiting PR value at about 30 cm depth. Therefore graphical representation of PR as a function of depth such as the one shown in Figure 5 is very informative. Nevertheless, this graph relates only to one fixed location. Such plots need to be generated for all coordinates, from which data have been collected across the field. More insight, however, could be gained by examining the distribution of PR rather than inspecting individual coordinates, requiring a 2D graphical representation.

Measured quantities at each location in the field were averaged over five measurements to create eight layers from 0 to 40 cm (0-5 cm, 5-10 cm, …, 35-40 cm), and a 2D soil compaction map was generated for each layer as shown in Figure 6 where only four out of eight layers were shown for simplicity. The soil compaction in these soil layers were marked with different color scales. The designed color scale depends upon the minimum and maximum measured PR values in the corresponding layer. In Figure 5a, variation from one grid to another did not seem to be important. As the depth increased, however, the variation within the same layer became more apparent, implying more random fluctuations in PR values across the field. The 2D graph of soil compaction becomes useful at this point in that coordinate tagged PR values make it possible especially to determine regions that shall be considered for deep tillage to break the hardpan. The drawback of 2D graphical representation hence is the need for creating various layers to be examined so that all problem areas could be identified.

**Figure 7.**
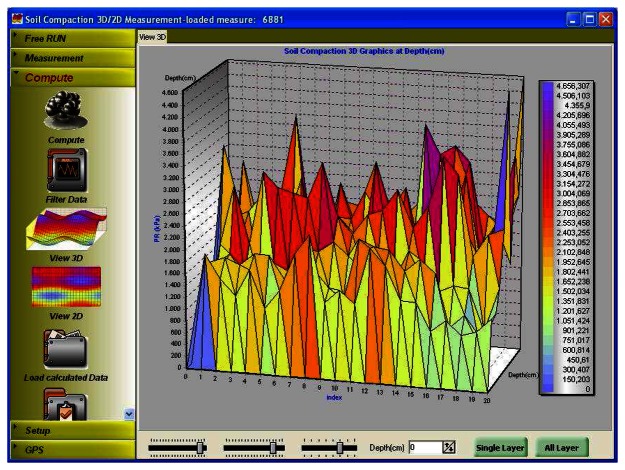
3D graphical display of penetration resistance across a defined area in a field.

3D graphics of soil compaction might provide a visual observation for compacted soil zones without the need for creating multiple graphics (Figure 7). Such a graph not only shows the distribution of penetration resistances over the field but displays the magnitude of penetration as a function of depth. Figure 7 shows all data points at all depths in the experimental site. It can be observed that penetration resistances at some locations are above the threshold of 2 MPa. 3D graph of PR distribution seems handy in that specific regions exposed to compaction greater than the threshold could be easily monitored by examining a single graphical display.

The system can incorporate GPS data to generate geo-referenced mapping. GPS, however, was not used in the field experiments because the measured coordinates were too close to differentiate between adjacent locations using an ordinary positioning system. GPS recordings not only provides necessary data for geo-referenced mapping but also establishes a baseline to mark coordinates so that temporal variations could be determined by acquiring data from the same locations at different times. Once compacted layers and depths have been identified, for instance, site-specific soil tillage can be practiced. The system tested in this study, however, was not designed for guiding variable-rate applicators, but can be utilized as a decision support system to determine spatial variations of penetration resistance.

## 4. Conclusions

The followings could be concluded as a result of this study:

- The penetrometer mounted on the three-point hitch of the tractor was driven by the tractor hydraulics. The system increased the speed of data collection and hence increased the field efficiency significantly compared to hand-held penetrometers.
- Spatial variations in soil penetration resistance make it difficult to accomplish a constant penetration speed using hand-held penetrometers, resulting in random errors that can not be eliminated. The operator errors were eliminated in this study as result of a standard penetration speed of 3 cm s^-1^.
- The software used in this study incorporates position data using a GPS receiver and can be used either to tabulate soil compaction data or plot graphs in 2D or 3D, providing immediate visual insight on the level of spatial variation of soil compaction.
- The instant geo-referenced compaction graphing can reduce post-processing time of penetration resistance data and could be used to determine which areas need different tillage treatments.
- Future studies on this system shall focus on mapping problem areas (locations suffering from compaction with PR>2 MPa) and then try to manage different zones accordingly with DPGS guided variable rate tillage equipment.
